# Plasmacytoma of the trachea: a surprising diagnosis

**DOI:** 10.1590/0100-3984.2015.0112

**Published:** 2017

**Authors:** Márcio Luís Duarte, Mariana Carneiro Barbosa de Brito, Fabricius André Lyrio Traple, José Luiz Masson de Almeida Prado, Luiz Carlos Donoso Scoppetta

**Affiliations:** 1 WebImagem, São Paulo, SP, Brazil.; 2 Hospital São Camilo, São Paulo, SP, Brazil.

**Figure 1:**
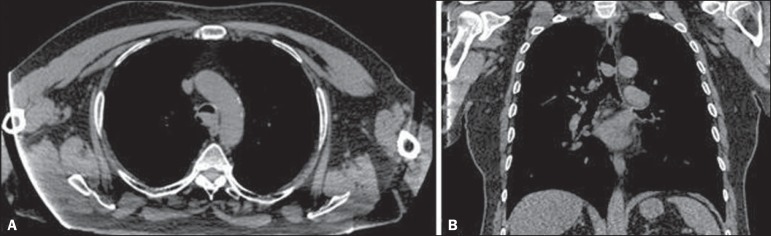
**A:** Axial CT scan, without contrast, showing an extensive, well-defined nodular mass in the distal trachea, measuring 2.1 × 1.3 × 1.7 cm, without signs of tracheal wall invasion. **B:** Coronal CT scan, without contrast, showing an expansive, well-defined nodular mass in the distal trachea, at the level of the carina, without signs of tracheal wall invasion.

**Figure 2:**
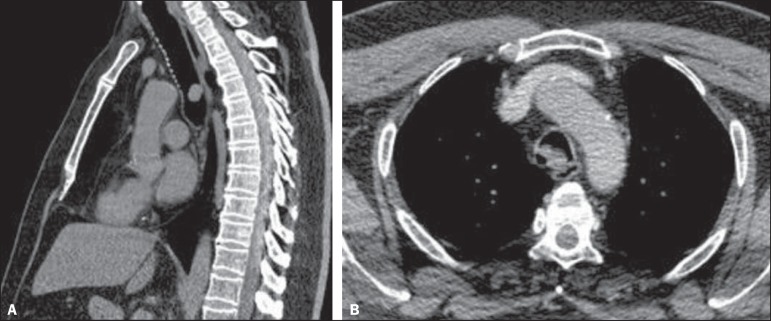
**A:** Sagittal CT scan (ROI: 51 HU), without contrast, showing an expansive, well-defined nodular mass in the distal trachea, without signs of tracheal wall invasion. **B:** Contrast-enhanced axial CT scan (ROI: 61 HU) showing an expansive nodular mass with no contrast uptake.

Dear Editor,

A 68-year-old man presented with a complaint of dyspnea on moderate exertion, and physical examination revealed stridor. The patient reported having previously been treated for chronic obstructive pulmonary disease and adenocarcinoma of the prostate, the latter having been treated with 39 radiotherapy sessions. He was a former smoker with a smoking history of 150 pack-years (3 packs/day for 50 years), having quit 4 years prior. We performed contrast-enhanced computed tomography (CT) of the neck and chest, which showed an expansive, well-defined nodular mass in the distal trachea, near the carina, without enhancement or signs of invasion of the tracheal walls (Figures 1 and 2). Bronchoscopy was requested for tumor resection, and symptom resolution was observed after the resection. The histopathological study identified an outer layer with the of appearance of plasmacytoid cells, sometimes with a central eosinophilic nucleolus—"cartwheel appearance"—and hyaline intracytoplasmic inclusions suggestive of Russell bodies. The immunohistochemical profile demonstrated positivity for CD3, CD20, CD45, CD56, kappa light chain, and CD138 in plasmacytes. In the context of the clinical status and test results, the findings were consistent with solitary extramedullary plasmacytoma.

Diseases involving the trachea or the main bronchi are not common^(1-4)^. Less common still are tracheal tumors, which account for only 1-2% of all respiratory tract tumors^(5,6)^, affecting mainly the lower third of the tract^(7)^. Such tumors can be locally invasive^(3)^, adenoid cystic carcinoma and squamous cell carcinoma being the malignant tumors most often affecting the trachea^(5,6,8)^. The most common symptoms are related to airway obstruction, dyspnea being the most common, and become more evident when the tracheal lumen is narrowed by more than 75%^(5,9)^. Other symptoms include cough, dysphonia, hoarseness, hemoptysis, stridor, dysphagia, nasal obstruction, epistaxis, rhinorrhea, ear pain, weight loss, and cyanosis^(6)^.

Extramedullary plasmacytoma of the trachea is a rare plasma cell malignancy (accounting for only 4% of plasma cell tumors), having been described in soft tissues outside the bone marrow, involving the submucosal lymphoid tissue, and occurring at different locations, especially in the upper airways, most often in the paranasal sinuses or nose^(5,8,10)^. Involvement of the larynx, hypopharynx, cervical glands, esophagus, cervical lymph nodes, middle ear, and mastoid is rare^(5)^, and tracheal involvement is even rarer^(5,11-13)^, occurring in only 3% of all extramedullary plasmacytomas^(9)^. As of 2005, only 15 cases of solitary extramedullary plasmacytoma of the trachea had been reported in the medical literature^(8)^. It primarily affects men between 50 and 60 years of age, with a male/female ratio ranging from 3:1 to 5:1^(5,8)^. Progression to multiple myeloma is considerably less frequent than is solitary plasmacytoma of the bone^(8)^.

In ultrasound of the neck, tracheal lesions, especially those located anteriorly, can be visualized clearly^(10)^. A CT scan allows the lumen, airway wall, and mediastinal structures to be evaluated. Multiplanar reconstructions are useful for assessing the type, degree, and longitudinal extent of the airway narrowing as well as the location of the tumor and its distance from the cricoid cartilage and carina^(5,7)^. Bronchoscopy correlates well with CT and can be used in order to resect the lesion^(5,7)^. The diagnosis is made through histological and immunohistochemical studies^(5,14)^. There was one reported case in which the tracheal plasmacytoma was identified as an incidental finding on positron emission tomography/CT^(5)^. The treatment can be surgical resection alone, radiotherapy alone, requiring annual monitoring, or a combination of the two^(5,13,14)^. There is local recurrence in approximately 30% of cases and metastasis in 15-40%^(13)^.
